# The extended pink esthetic score (E-PES): a reliability and multidirectional score-behavior study for peri-implant esthetic assessment

**DOI:** 10.1186/s40729-026-00705-2

**Published:** 2026-07-31

**Authors:** Hani ElNahass, Suzy N. Naiem, Bilal Al Nawas, Omnia K. Tawfik

**Affiliations:** 1 Present Address: Department of Periodontology, Faculty of Dentistry, Cairo University, Cairo, Egypt; 2 Head of Oral and Maxillofacial Surgery, Plastic Surgery Department, Mainz University, Mainz, Germany

**Keywords:** Dental implants, Peri-implant esthetics, Pink esthetic score, Esthetic assessment, Emergence profile, Mucosal scarring, Reliability, Multidirectional assessment

## Abstract

**Objectives:**

The Pink Esthetic Score (PES) is widely used to assess peri-implant soft-tissue esthetics around single-tooth implant restorations; however, frontal-view assessment may incompletely capture three-dimensional peri-implant features such as buccolingual contour, emergence profile, and mucosal scarring. This study introduced the Extended Pink Esthetic Score (E-PES), a multidirectional assessment framework incorporating frontal, profile, and occlusal views.

**Materials and methods:**

Thirty single-tooth implant restorations in the maxillary esthetic zone were evaluated using standardized photographs. Twenty blinded evaluators (five periodontists, five prosthodontists, five orthodontists, and five general practitioners) scored each case using two assessment systems. The traditional frontal-view assessment using the seven-variable PES, then the multidirectional E-PES assessment using frontal, profile, and occlusal views with nine variables. The E-PES retained the seven core PES variables, with emergence profile and mucosal scarring added to the variables to give a multidirectional view. Assessments were repeated after a three-week washout period with randomized image order. Reliability, agreement, and score-distribution analyses were performed.

**Results:**

A total of 600 first-round assessments were completed for each scoring system and included in the paired frontal-view versus multidirectional score analysis. Original PES and multidirectional E-PES-compatible total scores averaged 9.98 ± 3.96 and 9.76 ± 3.52, respectively, yielding a small but statistically significant mean paired difference of 0.22 points (*P* < 0.001). Scores differed in 442 of 600 paired assessments (73.67%). Agreement between frontal-view and multidirectional total scores remained excellent (ICC = 0.919; ICC = 0.958). The alveolar process/buccal contour parameter showed the greatest mean shift, decreasing from 1.31 ± 0.69 to 1.10 ± 0.74 (*P* < 0.001). Inter-examiner reliability for multidirectional total scores was moderate at the individual-rater level (single-measure ICC = 0.633) and high at the group level (average-measure ICC = 0.972).

**Conclusions:**

E-PES preserved the reliability of the original PES while identifying clinically relevant esthetic differences not fully captured by frontal-view assessment alone. Multidirectional evaluation particularly enhanced assessment of alveolar-process/buccal-contour morphology, supporting E-PES as a practical refinement of PES for more comprehensive peri-implant esthetic evaluation.

**Supplementary Information:**

The online version contains supplementary material available at 10.1186/s40729-026-00705-2.

## Introduction

Implant therapy has become a predictable treatment modality, with high survival rates and reliable osseointegration. As a result, implant survival alone is no longer sufficient to define treatment success, particularly in the maxillary esthetic zone. Contemporary implant therapy must also achieve biological stability, functional integration, and natural esthetic harmony [2, 19].

Traditional implant success criteria have focused primarily on mechanical and biological outcomes, including implant stability, osseointegration, marginal bone loss, and implant survival [1, 14, 15, 18]. Although these outcomes remain essential, they do not fully evaluate the final esthetics of implant-supported restorations. In the anterior maxilla, patient satisfaction is strongly influenced by the appearance of the peri-implant soft tissues, the harmony of the mucosal margin, the presence of papillae, the soft tissue contour, and the natural emergence of the restoration [3, 5, 8].

Among the available esthetic indices the Pink Esthetic Score (PES), introduced by [8], remains one of the most widely used clinical indices for assessing peri-implant soft tissue esthetics around single-tooth implant restorations [7, 11, 16]. The original PES evaluates seven parameters: mesial papilla, distal papilla, soft tissue level, soft tissue contour, alveolar process deficiency, soft tissue color, and soft tissue texture. Each variable is scored using a 0–1-2 ordinal scale, resulting in a maximum score of 14 points [8].

Despite its widespread use and clinical simplicity, the original PES has limitations in contemporary prosthetically driven implant dentistry. First, the original PES is primarily based on frontal-view assessment. This creates a false impression of esthetic success when buccolingual contour deficiencies, ridge collapse, or emergence profile discrepancies are not fully visible from the frontal perspective. Second, the original PES does not directly assess the emergence profile, although the transition between the implant restoration and peri-implant mucosa is central to natural esthetic integration [4, 10, 17, 22]. Third, mucosal scarring is not evaluated independently, even though scar-related fibrotic lines, tissue contraction, color alteration, and contour irregularities may compromise esthetic outcomes [6].

Peri-implant esthetics are inherently three-dimensional. A single frontal photograph may be insufficient to evaluate buccal contour, soft tissue volume, cervical convexity, or the relationship between the crown and surrounding mucosa. Profile and occlusal views may reveal deficiencies that are camouflaged in frontal photographs. Accordingly, a multidirectional photographic protocol may provide a more complete clinical representation of the peri-implant esthetic condition [3, 6].

The Extended Pink Esthetic Score was developed to address these limitations while preserving the simplicity of the original PES. The E-PES retains the seven original PES variables and adds two clinically relevant parameters: emergence profile and mucosal scarring. It also incorporates frontal, profile, and occlusal photographic views to improve assessment of three-dimensional peri-implant esthetics.

The aim of the present study was to introduce and preliminarily evaluate the E-PES as a multidirectional peri-implant esthetic assessment framework for single-tooth implant restorations in the maxillary esthetic zone and to investigate whether multidirectional assessment provides additional clinically relevant information while maintaining reproducible total-score behavior.

## Materials and methods

### Study design and ethical approval

This observational clinical multidirectional score-behavior study evaluated the E-PES framework for single-tooth implant restorations in the maxillary esthetic zone. The investigation was designed to assess whether a standardized multidirectional photographic protocol combined with dedicated scoring for emergence profile and mucosal scarring provides additional reproducible esthetic information beyond frontal-view assessment.

Ethical approval for the current study was granted by the Ethics Committee of the Faculty of Dentistry, Cairo University, Egypt (Approval Number: 29-2-23). All participating patients provided written informed consent for acquisition, analysis, and publication of their clinical photographs.

### Case selection and photographic protocol

Thirty single fully restored maxillary implant-supported restorations in the esthetic zone were included. All restorations had been delivered at least one year before assessment. Cases were selected using purposive sampling to ensure representation of a broad spectrum of esthetic outcomes, ranging from highly esthetic to compromised clinical presentations. Cases with incomplete or insufficient-quality photographs, multiple adjacent implants, or restorations outside the maxillary esthetic zone were not included.

**Fig. 1:**
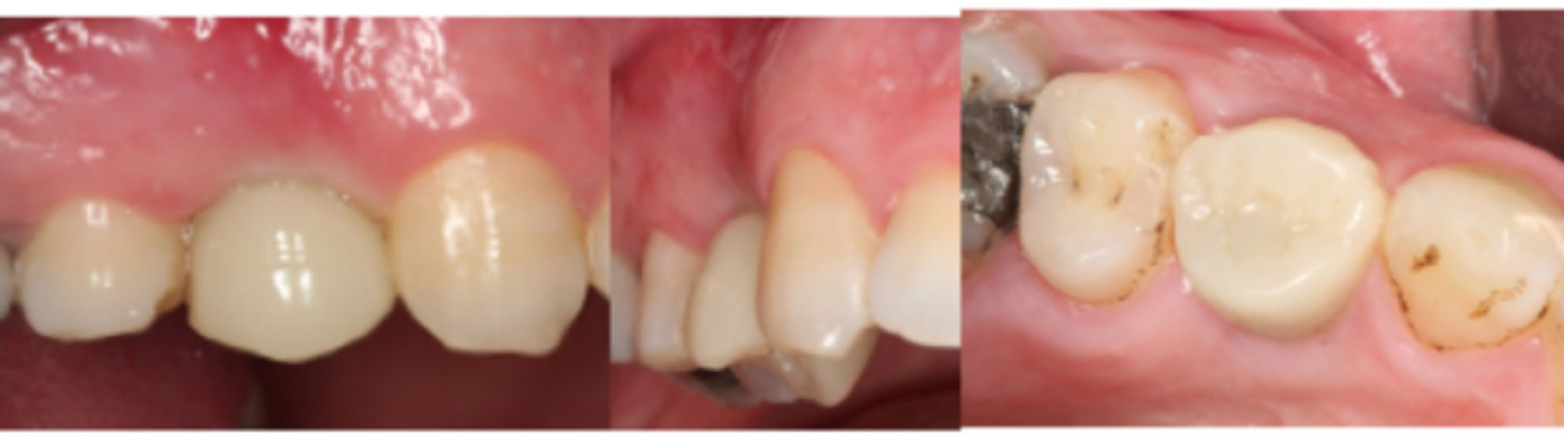
Standardized multidirectional photographic protocol showing (A) frontal, (B) profile, and (C) occlusal views used for E-PES assessment. The figure illustrates the additional perspectives incorporated into E-PES compared with frontal-view PES assessment

Each case was documented using standardized clinical photographs. Three views were obtained for each implant restoration: frontal, profile, and occlusal. All photographs were taken using a Canon EOS 600D digital camera equipped with a Canon Macro Ring Lite MR-14EX II flash system. Photographs were standardized for angulation, distance, magnification, and patient positioning as far as clinically possible. The frontal view was used for the original PES assessment, while frontal, profile, and occlusal views were used for E-PES assessment. The standardized multidirectional photographic protocol is illustrated in Fig. 1.

### Evaluator panel and calibration protocol

A multi-specialty panel of 20 independent blinded evaluators was assembled. The panel included five periodontists, five prosthodontists, five orthodontists, and five general dental practitioners. Before formal scoring, evaluators completed a calibration session led by the principal investigator. The evaluators received a reference manual containing operational definitions, scoring criteria, and visual examples for both the original PES and the E-PES. Calibration was performed using five non-study implant cases that were excluded from the final analysis. Calibration was considered acceptable when the panel achieved an ICC greater than 0.80 for total-score agreement on the calibration set.

### Scoring systems and matrix definitions

The original PES core evaluates seven soft-tissue parameters: mesial papilla, distal papilla, soft-tissue margin level, soft-tissue contour, alveolar process/buccal contour, soft-tissue color, and soft-tissue texture. The multidirectional E-PES expands this matrix to nine variables, preserving the seven core PES components and adding emergence profile and mucosal scarring. Both the E-PES and the PES use the same 3-point scoring system (0, 1, 2), with scores assigned according to the degree of fulfillment of each esthetic criterion (0 = absent/not present, 1 = partial, 2 = complete). In the multidirectional E-PES assessment, the nine variables were scored using frontal, profile, and occlusal views. The profile view specifically supported emergence-profile evaluation, and the occlusal view specifically supported buccolingual/alveolar-contour evaluation. Mucosal scarring was assessed independently from general soft-tissue texture and focused on visible fibrotic lines, localized contraction, scar-related color alteration, and contour irregularities. The total E-PES score ranges from 0 to 18, compared with a maximum score of 14 for the original PES, reflecting the inclusion of two additional assessment variables while maintaining the same 0–2 scoring system for each variable. (E-PES scoring matrix Table 1.)

**Table 1:** Extended pink esthetic score (E-PES) scoring matrix

Variable	Assessment criterion/principal view support	Score 0	Score 1	Score 2
Mesial papilla	Shape vs. contralateral natural tooth reference	Absent	Incomplete/partial	Complete
Distal papilla	Shape vs. contralateral natural tooth reference	Absent	Incomplete/partial	Complete
Soft-tissue margin level	Vertical position relative to natural tooth reference	Major discrepancy (> 2 mm)	Minor discrepancy (1–2 mm)	No discrepancy (< 1 mm)
Soft-tissue contour	Natural architecture matching reference tooth	Unnatural/distorted	Fairly natural	Highly natural
Alveolar process / buccal contour	Buccal contour; frontal view in baseline, occlusal-view support in multidirectional assessment	Severe depression/ridge collapse	Mildly depressed or overcorrected contour	Ideal, harmonious buccal contour
Soft-tissue color	Color match vs. reference natural tissue	Obvious clear mismatch	Moderate/borderline difference	No perceptible difference
Soft-tissue texture	Surface character/stippling vs. reference tissue	Obvious clear mismatch	Moderate/borderline difference	No perceptible difference
Emergence profile (new)	Peri-implant mucosal transition; frontal view in baseline, profile-view support in multidirectional assessment	Concavity, severe step, or gross overbulking	Minor flattening or slight crown-mucosa mismatch	Smooth convex transition harmonious with adjacent tissues
Mucosal scarring (new)	Visible surgical scar or fibrotic alteration; assessed separately from soft-tissue texture	Pronounced scar, contraction, or contour deformity	Localized linear scar or mild fibrotic line without harmony loss	No visible fibrotic lines or contraction

### Assessment protocol

Each evaluator assessed all 30 cases using the standard PES assessment approach based on frontal-view photography, followed by reassessment using the extended E-PES framework incorporating additional variables and photographic information. This paired design allowed evaluation of the impact of the additional E-PES components and photographic perspectives while maintaining consistency in the scoring methodology. To assess test–retest intra-examiner reproducibility, all evaluations were repeated after a three-week washout period, with case order randomized to minimize recall bias. Evaluators remained blinded to patient identifiers and clinical information throughout the study and completed a questionnaire regarding the perceived value of the additional photographic views and E-PES parameters.

### Statistical analysis

Statistical analyses were performed using IBM SPSS Statistics version 26.0 (IBM Corp., Armonk, NY, USA) and Python 3.11 (SciPy, statsmodels, NumPy, pandas, and Matplotlib). Continuous variables are presented as mean ± standard deviation.

Differences between original PES and multidirectional E-PES total scores were evaluated using paired comparisons. The relationship between both assessment conditions was assessed using Pearson correlation analysis. Bland–Altman analysis was performed to characterize systematic score shifts between the frontal-view and multidirectional conditions. Inter-examiner agreement and test–retest intra-examiner reproducibility were treated as separate reliability constructs. Inter-examiner agreement refers to agreement among evaluators within an assessment condition, whereas test–retest intra-examiner reproducibility refers to stability of the same evaluator across the repeated washout round.

A linear mixed-effects model was fitted with total score as the dependent variable, assessment condition and evaluator specialty as fixed effects, and random intercepts for case and evaluator. Variance decomposition was performed to estimate the relative contributions of case-related and evaluator-related variability. Statistical significance was established at *P* < 0.05.

## Results

A total of 600 first-round assessments were completed per assessment condition. For paired frontal-to-multidirectional score-behavior analyses, 600 complete paired observations were available and are used consistently for the reported score-change frequencies. Because assessments were repeated after the washout period, 1200 assessments were completed per condition and 2400 scoring events overall. (Table 2).

**Table 2:** Specialty comparison of score behavior between frontal-view PES-compatible assessment and multidirectional E-PES assessment (complete paired-analysis set; n = 600)

Evaluator specialty	Paired assessments (n)	Frontal-view PES-compatible score (mean ± SD)	Multidirectional E-PES-compatible score (mean ± SD)	Paired score changes, n (%)
Periodontists	150	10.70 ± 3.40	10.32 ± 2.97	104 (69.3%)
Prosthodontists	150	9.70 ± 3.96	9.46 ± 3.71	108 (72.0%)
General practitioners	150	10.39 ± 3.83	10.18 ± 3.44	113 (75.3%)
Orthodontists	150	9.13 ± 4.40	9.07 ± 3.81	117 (78.0%)
Overall	600	9.98 ± 3.96	9.76 ± 3.52	442 (73.7%)

^**^PES-compatible scores represent the seven shared PES variables assessed under both conditions (score range 0–14). Multidirectional E-PES assessment additionally included emergence profile and mucosal scarring as separate variables; the full E-PES score range is 0–18

### Comparison of PES and E-PES scores and effect of multidirectional assessment

**Fig. 2:**
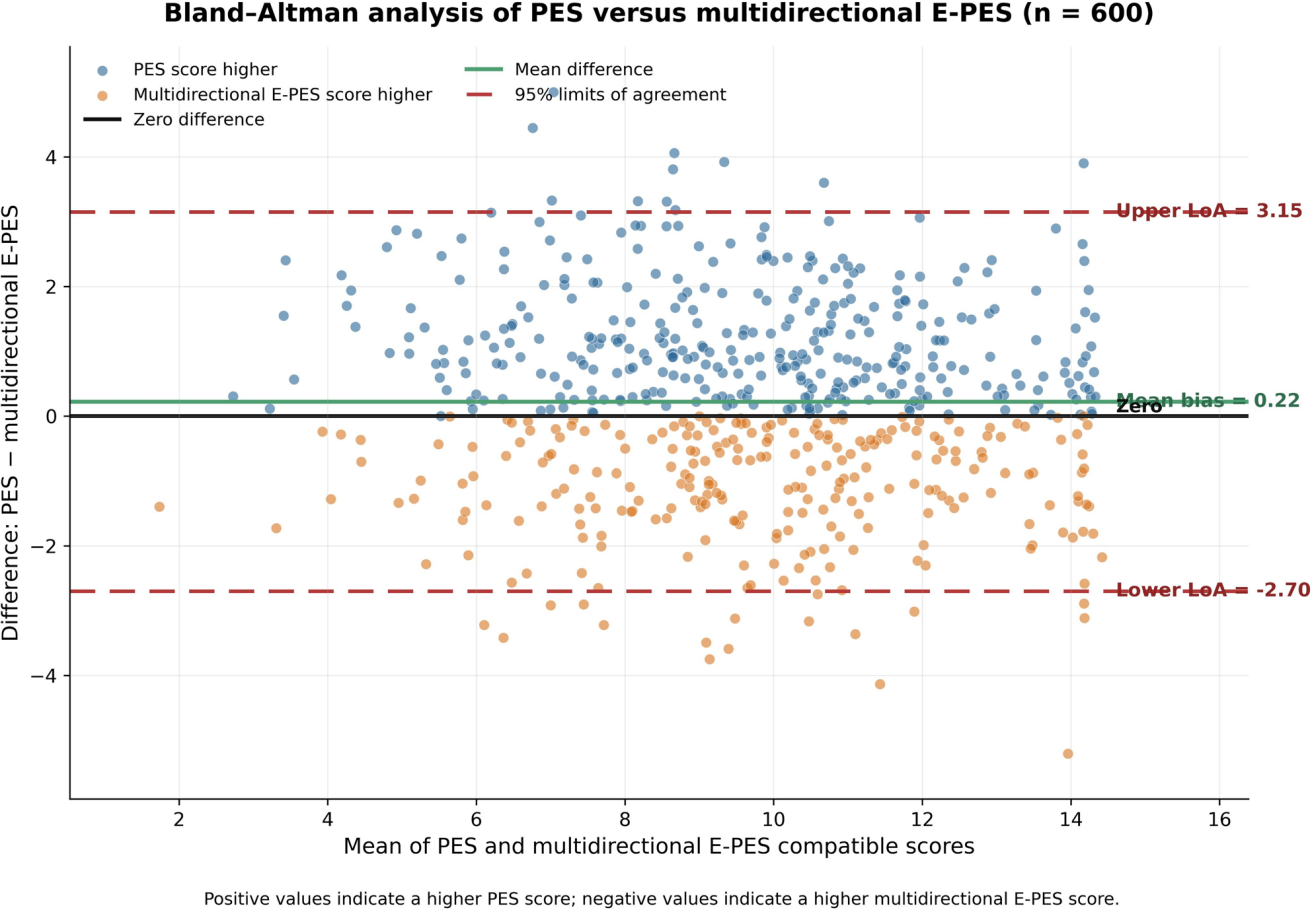
Bland–Altman analysis comparing PES-compatible total scores obtained from frontal-view assessment with multidirectional E-PES-compatible total scores in the complete paired-analysis set (n = 600). Each point represents one paired assessment. Positive differences indicate higher PES scores, whereas negative differences indicate higher multidirectional E-PES scores. The solid green line represents the mean difference between methods (PES minus multidirectional E-PES = 0.22 points), the dashed red lines indicate the 95% limits of agreement (− 2.70 to 3.15), and the solid black line marks zero difference. Blue points indicate assessments in which the PES score was higher, orange points indicate assessments in which the multidirectional E-PES score was higher, and grey points indicate identical scores. The analysis describes systematic score behavior between the two assessment approaches and is not intended to establish method interchangeability

Across the 600 complete paired observations, mean total scores decreased slightly from 9.98 ± 3.96 under frontal-view assessment to 9.76 ± 3.52 under multidirectional E-PES assessment. The mean paired difference (frontal minus multidirectional) was 0.22 points (*P* < 0.001), indicating that multidirectional assessment altered esthetic perception. Despite this difference, frontal-view and multidirectional total scores remained strongly correlated (r = 0.913), suggesting that E-PES modifies score behavior by adding clinically relevant views rather than measuring an unrelated construct. Figure 2.

**Fig. 3:**
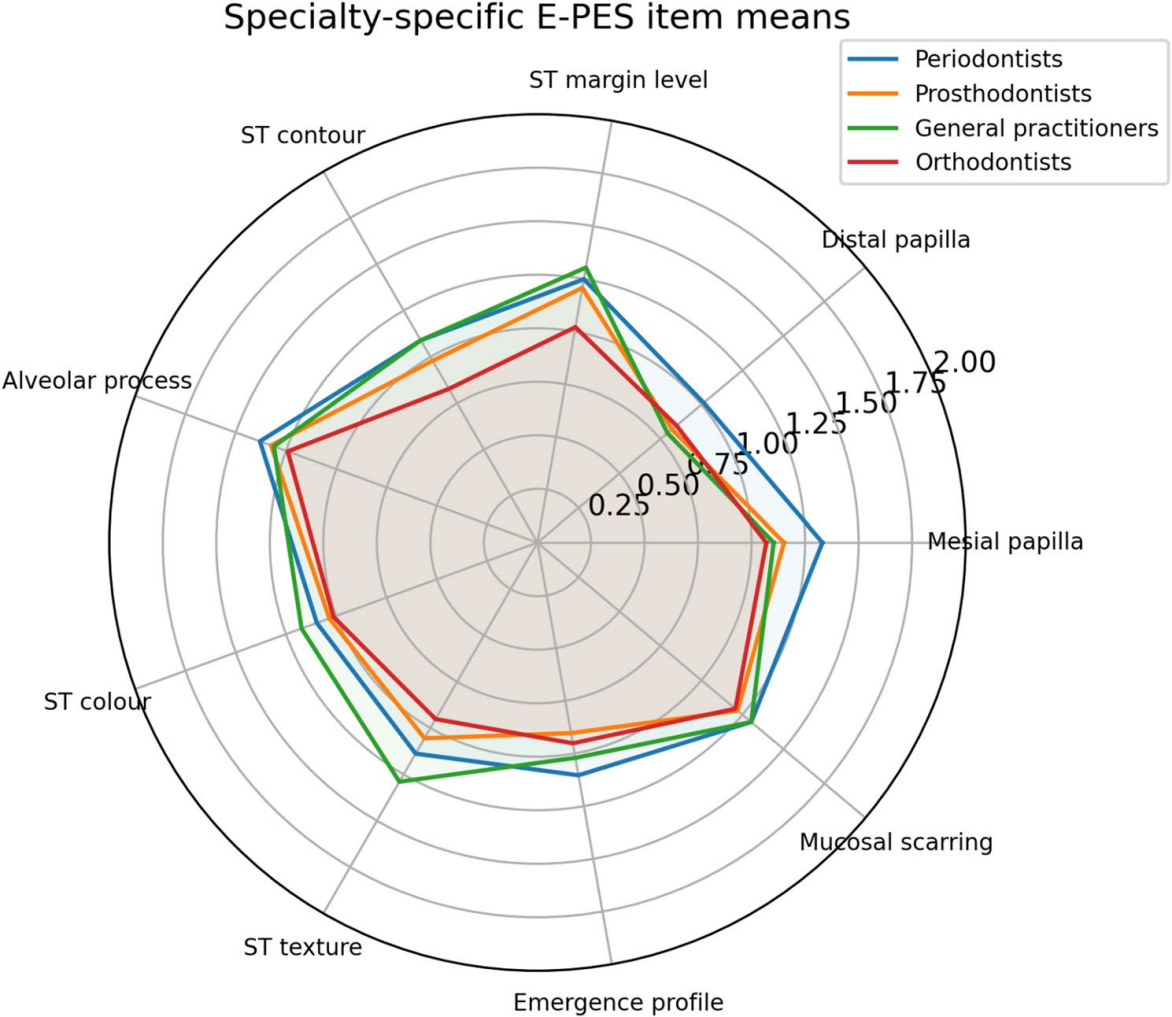
Specialty-specific mean item scores across the nine variables of the multidirectional E-PES assessment (n = 600 evaluator-case assessments; item score range, 0–2). The radar plot illustrates exploratory differences in mean scoring patterns among periodontists, prosthodontists, general practitioners, and orthodontists for each E-PES variable, including mesial papilla, distal papilla, soft-tissue margin level, soft-tissue contour, alveolar process/buccal contour, soft-tissue color, soft-tissue texture, emergence profile, and mucosal scarring. The figure represents specialty-related scoring patterns within the multidirectional E-PES assessment and does not indicate differences in overall E-PES validity between specialties

**Fig. 4:**
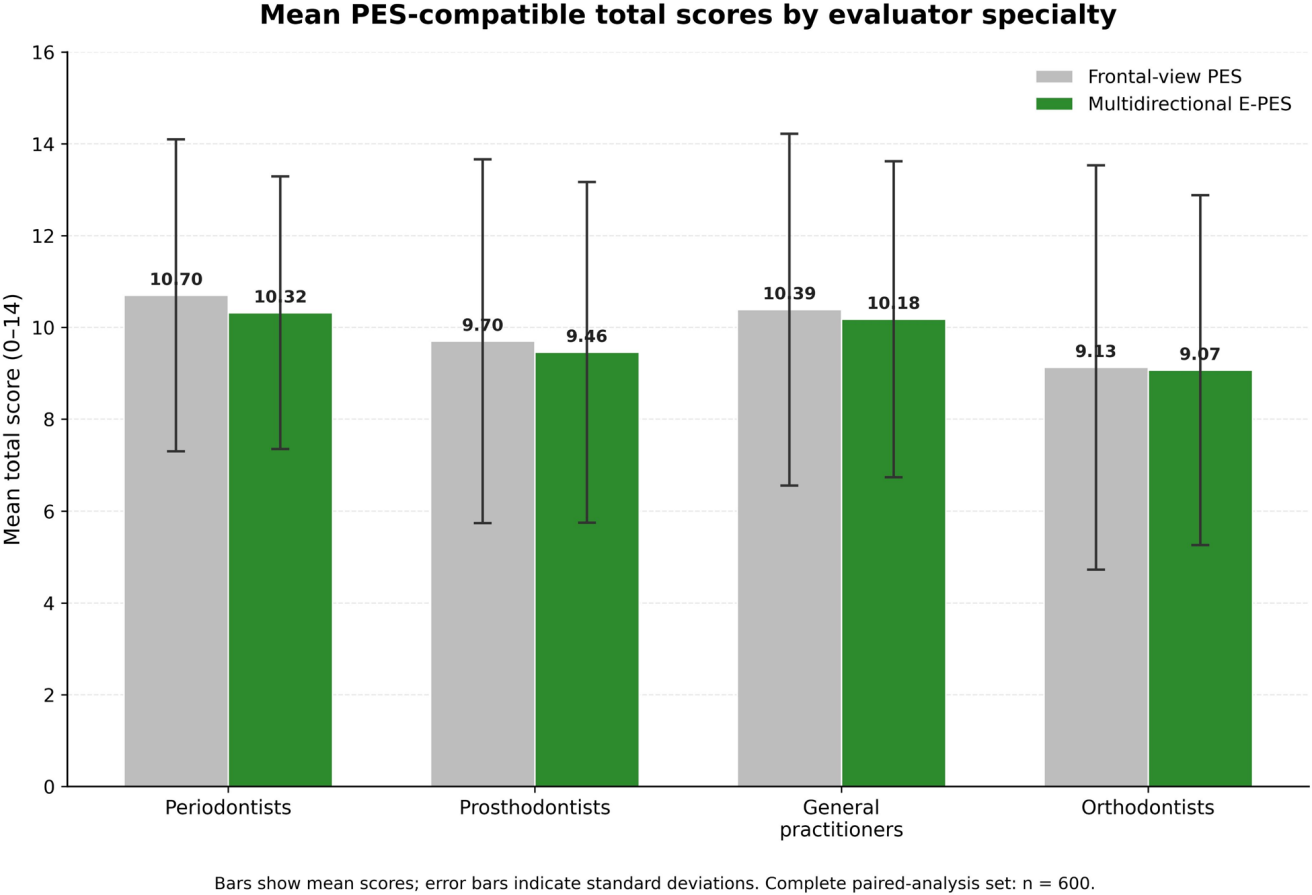
Mean PES-compatible total scores (seven shared variables; score range, 0–14) according to evaluator specialty under frontal-view PES-compatible assessment and multidirectional E-PES assessment (complete paired-analysis set; *n* = 600). Bars represent mean scores and error bars represent standard deviations (SD). The multidirectional assessment incorporated additional photographic views, while total scores shown here are restricted to the seven variables shared between the original PES and E-PES to permit direct comparison

Total scores changed following multidirectional assessment in 442 of 600 complete paired observations (73.67%). By specialty, score changes occurred in 69.0% of complete paired evaluations among periodontists, 71.7% among prosthodontists, 75.2% among general practitioners, and 78.6% among orthodontists. Significant differences among specialties were observed for both frontal-view total scores (*p* = 0.003) and multidirectional E-PES total scores (*p* = 0.006), although these findings should be interpreted cautiously because each specialty group included only five evaluators. Specialty-specific item profiles and total-score comparisons are shown in Figs. 3 and 4, respectively.

Multidirectional assessment was associated with reduced case-level variability, with mean inter-rater standard deviation decreasing from 2.51 under PES to 2.11 under E-PES, suggesting improved consistency among evaluators.

### Item-level score behavior

**Fig. 5:**
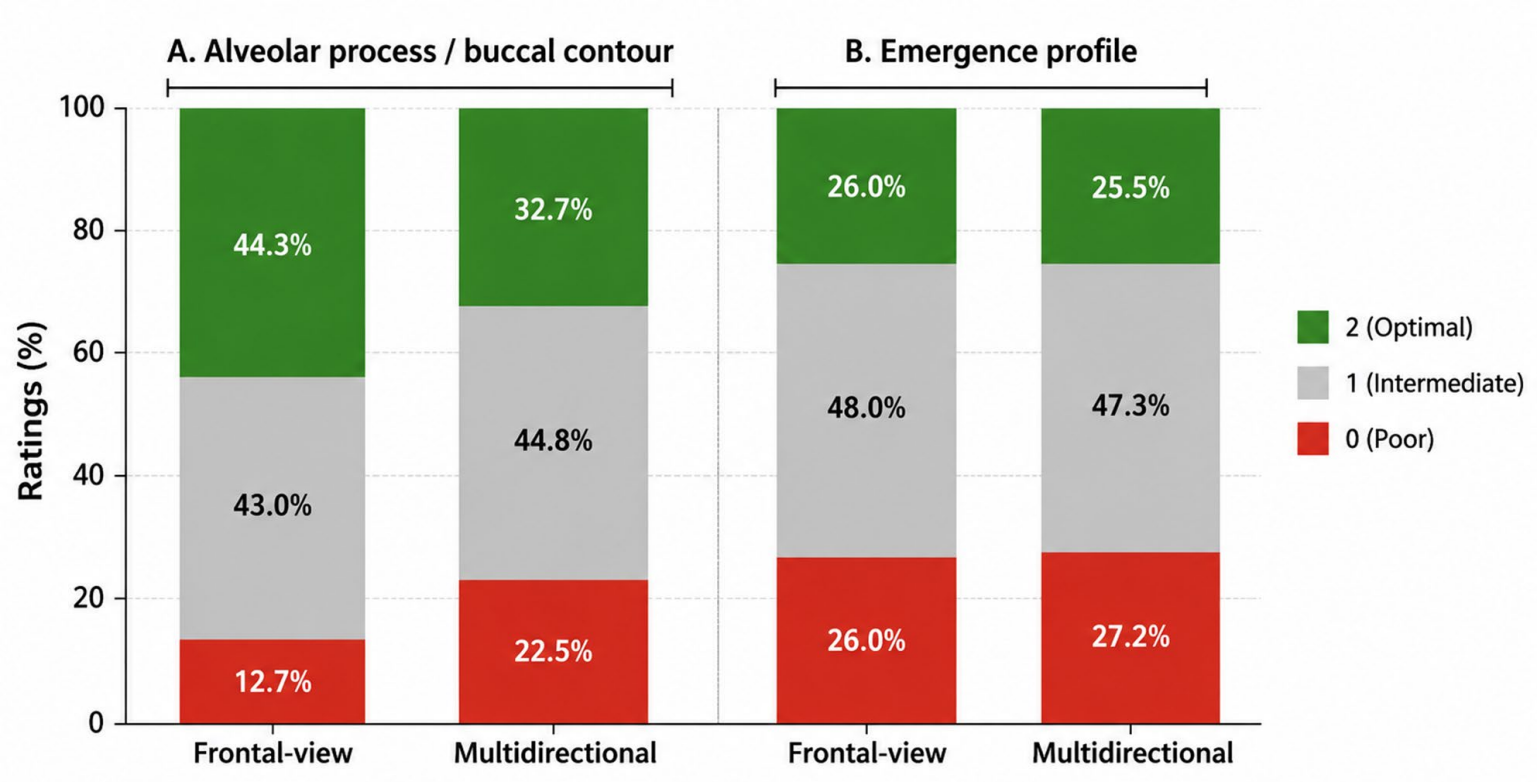
Distribution of ordinal ratings (scores 0–2) for alveolar process/buccal contour and emergence profile under frontal-view and multidirectional E-PES assessment (complete paired-analysis set; *n* = 600 paired evaluations). Stacked bars represent the percentage of ratings assigned to each ordinal category. Multidirectional assessment shifted alveolar process/buccal contour ratings toward lower scores, indicating improved detection of buccal contour deficiencies after incorporating occlusal-view information, whereas emergence-profile score distributions remained largely unchanged

The alveolar process/buccal contour score decreased from 1.31 ± 0.69 under frontal-view assessment to 1.10 ± 0.74 after occlusal-supported multidirectional evaluation (Wilcoxon *p* < 0.001; Bowker *p* < 0.001). In contrast, emergence profile demonstrated no significant mean shift (0.99 ± 0.72 to 0.98 ± 0.73; Wilcoxon *p* = 0.870; Bowker *p* = 0.089). Distributional analysis showed that multidirectional assessment increased the frequency of lower alveolar process scores, suggesting that occlusal views revealed contour deficiencies that were less apparent from frontal photographs alone. Low item-level kappa values for alveolar process/buccal contour and emergence profile should therefore be interpreted cautiously; they may reflect view-dependent scoring after additional views were introduced, but they may also indicate remaining ambiguity in the scoring criteria. The paired item-level Table 3, and the ordinal rating distributions are shown in Fig. 5.

**Table 3:** Paired frontal-view to multidirectional score behavior for view-sensitive E-PES variables (complete paired-analysis set; n = 600; item score range, 0–2)

Variable	Frontal-view/Original PES mean ± SD	Multidirectional E-PES mean ± SD	Ratings changed	Wilcoxon P	Bowker P	Mean weighted kappa (95% CI)
Alveolar process/buccal contour	1.31 ± 0.69	1.10 ± 0.74	55.3%	< 0.001	< 0.001	0.15 (0.08–0.21)
Emergence profile	0.99 ± 0.72	0.98 ± 0.73	55.5%	0.870	0.089	0.17 (0.10–0.25)

### Reliability and agreement

Pairwise agreement between frontal-view and multidirectional total scores was high (ICC = 0.919 for single measurements and ICC = 0.958 for average measurements), indicating that both assessment approaches produced highly consistent overall scores. These ICCs reflect agreement between the two assessment conditions and should not be interpreted as measures of intra-examiner repeatability. Inter-examiner reliability was moderate for individual raters but high for average ratings in both the frontal-view assessment (single-measure ICC = 0.593; average-measure ICC = 0.967) and the multidirectional E-PES assessment (single-measure ICC = 0.633; average-measure ICC = 0.972). Test–retest intra-examiner reliability from the three-week reassessment is reported separately. The main reliability outcomes are summarized in Table 4, and item-level inter-examiner Fleiss kappa values are reported in Table 5.

**Table 4:** Main reliability, agreement, and score-behavior outcomes. Inter-examiner ICCs, paired condition-consistency ICCs, and test–retest intra-examiner reproducibility are distinct reliability constructs; total score range, 0–18

Parameter/metric	Statistical index	Result	Interpretation
Paired condition consistency	ICC(2,1), frontal-view vs. multidirectional total score (not test–retest)	0.919	High paired total-score consistency between assessment conditions
Paired condition consistency	ICC(2,k), frontal-view vs. multidirectional total score (not test–retest)	0.958	High average-measure consistency between assessment conditions
Frontal-view total score	Inter-examiner single-measure ICC	0.593	Moderate individual-rater inter-examiner agreement
Frontal-view total score	Inter-examiner average-measure ICC	0.967	High group-level inter-examiner reliability
Multidirectional E-PES total score	Inter-examiner single-measure ICC	0.633	Moderate individual-rater inter-examiner agreement
Multidirectional E-PES total score	Inter-examiner average-measure ICC	0.972	High group-level inter-examiner reliability
Frontal-view E-PES-compatible matrix	Cronbach alpha	0.769	Supplementary matrix consistency
Multidirectional E-PES matrix	Cronbach alpha	0.670	Supplementary matrix consistency
Alveolar process / buccal contour	Mean quadratic weighted kappa	0.15 (0.08–0.21)	Low item-level agreement; may reflect view-dependent scoring and residual scoring ambiguity
Emergence profile	Mean quadratic weighted kappa	0.17 (0.10–0.25)	Low item-level agreement; may reflect view-dependent scoring and residual scoring ambiguity
Exploratory Bland–Altman score behavior	Mean paired difference (frontal-view minus multidirectional); 95% limits of agreement	0.22 points; -2.70 to 3.15	Small systematic score shift; not interpreted as method interchangeability
Test–retest intra-examiner reproducibility	Dedicated ICC from repeated washout-round ratings	AUTHOR QUERY: insert calculated ICC value(s)	Must be reported separately from inter-examiner ICCs and paired condition-consistency ICCs

**Table 5:** Inter-examiner Fleiss kappa for item-level E-PES variables (item score range, 0–2; descriptive interpretation only)

Item	Fleiss kappa	Interpretation
Mesial papilla	0.350	Fair agreement
Distal papilla	0.343	Fair agreement
ST margin level	0.235	Fair agreement
ST contour	0.233	Fair agreement
Alveolar process	0.114	Slight agreement; interpret cautiously
ST colour	0.251	Fair agreement
ST texture	0.193	Slight agreement
Emergence profile	0.178	Slight agreement; interpret cautiously
Mucosal scarring	0.306	Fair agreement
Alveolar process/buccal contour (multidirectional)	0.307	Fair agreement; interpret cautiously
Emergence profile (multidirectional)	0.225	Fair agreement; interpret cautiously

Kappa interpretation follows conventional descriptive thresholds (< 0.20, slight; 0.21–0.40, fair; 0.41–0.60, moderate; 0.61–0.80, substantial; > 0.80, almost perfect). These labels are descriptive and should be interpreted cautiously because the ratings are clustered within cases and evaluators. Low kappa values for alveolar process/buccal contour and emergence profile may reflect both view-dependent scoring and remaining ambiguity in the item-level scoring criteria

Exploratory Bland–Altman analysis demonstrated a small mean difference of 0.22 points (frontal-view minus multidirectional score), with 95% limits of agreement ranging from − 2.70 to 3.15. Because the multidirectional E-PES incorporates additional photographic information, this analysis was intended to describe how scores changed between assessment methods rather than to evaluate interchangeability. The small positive bias indicates that incorporating multiple photographic views slightly altered esthetic scores while preserving a high level of overall agreement with the original frontal-view assessment.

### Global linear mixed-effects model

Global mixed-effects modelling demonstrated that multidirectional assessment resulted in significantly lower scores compared with frontal-view assessment (beta = − 0.222, *p* < 0.001). Specialty-related differences were observed, with prosthodontists and orthodontists assigning lower scores than periodontists, whereas no significant differences were detected between periodontists and general practitioners.

Variance partitioning demonstrated that case heterogeneity represented the largest source of variability (61%), followed by evaluator-related differences (31%), while residual unexplained variance accounted for only 7.9%. These findings indicate that score variation primarily reflected case-specific esthetic characteristics, although professional background also influenced esthetic perception. The complete mixed-effects model results are presented in Table 6.

**Table 6:** Global linear mixed-effects model variance partition and fixed effects

Component	Parameter/covariate	Estimate or variance	SE/share	Test statistic	*P* value
Fixed effect	Intercept (periodontist, frontal-view reference)	10.625	0.574	z = 18.51	< 0.001
Fixed effect	Specialty: prosthodontists	− 0.938	0.260	z = − 3.61	< 0.001
Fixed effect	Specialty: general practitioners	− 0.231	0.260	z = − 0.89	0.374
Fixed effect	Specialty: orthodontists	− 1.414	0.260	z = − 5.44	< 0.001
Fixed effect	Multidirectional vs. frontal-view	-0.222	0.062	z = − 3.59	< 0.001
Random effect	Case ID random intercept	8.545	61.0%	–	–
Random effect	Evaluator ID random intercept	4.340	31.0%	–	–
Residual	Unexplained variance	1.113	7.9%	–	–

## Discussion

The present study introduced and preliminarily evaluated the E-PES, a multidirectional framework for assessing peri-implant esthetics around single-tooth implant restorations. The E-PES was designed to preserve the simplicity of the original PES while addressing clinically relevant limitations related to three-dimensional assessment. Unlike the original PES, which relies primarily on frontal-view assessment of seven variables, the E-PES introduces two complementary modifications: multidirectional photographic assessment of the existing variables and independent assessment of two additional esthetic parameters—emergence profile and mucosal scarring—that are not explicitly represented in the original index.

The original PES remains one of the most widely used and clinically practical tools for evaluating peri-implant soft tissue esthetics. However, the present findings suggest that frontal-view assessment alone may incompletely characterize peri-implant esthetic outcomes. In this study, the addition of profile and occlusal views and the combined multidirectional assessment framework changed 442 of 600 complete paired observations (73.67%). This finding supports the concept that a single frontal photograph may create a false impression of esthetic success by masking buccolingual contour deficiencies, ridge collapse, emergence profile discrepancies, or mucosal scarring.

Exploratory Bland–Altman analysis demonstrated a small systematic shift between frontal-view and multidirectional assessment, with a mean paired difference of 0.22 points and 95% limits of agreement from − 2.70 to 3.15. Although the two conditions remained strongly correlated, multidirectional evaluation tended to generate slightly lower scores, suggesting increased sensitivity to esthetic deficiencies that may not be fully appreciated by frontal-view assessment alone. These findings support the concept that E-PES extends rather than replaces the original PES framework.

The inclusion of emergence profile is a central innovation of the E-PES. In contemporary prosthetically driven implant therapy, the emergence of the implant-supported crown through the peri-implant mucosa is essential for natural esthetic integration. A restoration may appear acceptable from the frontal view while still showing a deficient cervical contour or flattened buccal emergence when assessed from the profile view. By incorporating emergence profile into the score, the E-PES expands esthetic evaluation beyond soft tissue symmetry alone.

Although alveolar process/buccal contour and emergence profile were scored as separate variables, they represent closely related components of the three-dimensional peri-implant architecture. The explicit evaluation of emergence profile may have heightened evaluators’ awareness of the three-dimensional relationship between buccal contour and the prosthesis–mucosa transition, contributing to the greater score shift observed for alveolar process/buccal contour after multidirectional assessment. This relationship may also explain the relatively low weighted kappa values observed for these two variables. Rather than necessarily indicating poor reproducibility, the lower agreement may reflect the additional information provided by profile and occlusal views, which influenced evaluators’ perception of contour and emergence profile. Nevertheless, some ambiguity in the current scoring criteria cannot be excluded, and future refinements should include clearer visual reference standards and additional calibration examples to improve consistency in scoring these parameters.

Mucosal scarring was incorporated as an independent parameter because it represents a distinct esthetic characteristic that is not fully captured by the original soft-tissue texture variable. Although changes in soft-tissue texture may arise from biological restorative or surgical factors, visible scarring reflects the esthetic consequences of surgical wound healing, including fibrotic bands, localized contraction, contour irregularities, and scar-associated color changes. This distinction may be particularly relevant following simultaneous augmentation procedures, where acceptable tissue texture may coexist with clinically visible scarring. By assessing scarring separately, the E-PES aims to provide a more comprehensive evaluation of peri-implant soft-tissue esthetics [13, 20, 21].

The specialty-related differences observed in this study are clinically relevant but exploratory. Orthodontists assigned the lowest esthetic scores, whereas periodontists and general practitioners assigned higher scores. This may reflect differences in specialty-specific training and esthetic perception. Orthodontists are frequently trained to detect subtle deviations in tooth position, axial inclination, gingival margin symmetry, smile-line harmony, and micro-esthetic discrepancies. This may make them more critical of minor contour, symmetry, and emergence-profile deviations. Periodontists, in contrast, may place greater emphasis on soft tissue health, stability, and biological integration, potentially explaining their relatively higher ratings. Nevertheless, case-related variability exceeded evaluator-related variability, indicating that score differences were driven primarily by case characteristics rather than by specialty alone. Because each specialty group included only five evaluators, these specialty findings should be interpreted as hypothesis-generating rather than definitive [9, 12].

The present findings support the E-PES as a promising multidirectional assessment framework rather than a fully validated assessment index. Further validation is needed before the E-PES can be considered a definitive replacement for the original PES. Specifically, future studies should include larger multicenter samples, independent expert-panel classification, patient-reported esthetic outcomes, three-dimensional digital surface-scan analysis, and formal reporting of dedicated test–retest intra-examiner reproducibility values.

## Limitations

Several limitations should be acknowledged. First, although the E-PES incorporates frontal, profile, and occlusal photographic views, it remains based on static two-dimensional images. Therefore, subtle volumetric deficiencies, buccolingual ridge collapse, soft tissue thickness differences, and dynamic smile-related esthetic changes may still be incompletely captured.

Second, three-dimensional digital surface scans were not included. STL-based volumetric analysis or intraoral optical scanning may provide a more objective reference for future validation of peri-implant contour, emergence profile, and ridge volume.

Finally, patient-reported esthetic outcomes were not included. Clinician-based esthetic scores may not fully reflect patient perception, and future studies should correlate E-PES values with patient satisfaction and patient-reported esthetic acceptability.

## Conclusion

The E-PES represents a multidirectional, prosthetically driven extension of the original Pink Esthetic Score for evaluating peri-implant esthetics around single-tooth implant restorations. Incorporation of profile and occlusal photographs together with separate assessment of emergence profile and mucosal scarring altered esthetic perception while maintaining high total-score consistency with frontal-view assessment. Variance decomposition demonstrated that score variability was driven predominantly by case-related characteristics rather than evaluator identity, supporting their contribution to a more comprehensive peri-implant esthetic assessment. Further multicenter studies incorporating patient-reported outcomes, dedicated test–retest reproducibility analysis, and three-dimensional digital analyses are required for external validation.

## Supplementary Information

Below is the link to the electronic supplementary material.

Supplementary Material 1

Supplementary Material 1

## Data Availability

The item-level raw dataset supporting the findings of this study is available from the corresponding author upon reasonable request.

## Declarations

### Government declaration

None of the authors listed in the manuscript are employed by a government agency that has a primary function other than research and/or education. None of the authors are submitting this manuscript as an official representative or on behalf of a government.
