# Supplementary material for: The extended pink esthetic score (E-PES): a reliability and multidirectional score-behavior study for peri-implant esthetic assessment
Source: Int J Implant Dent. 2026 Jul 31;12:39. doi: 10.1186/s40729-026-00705-2 (PMC13424060; doi:10.1186/s40729-026-00705-2)
Supplement: Supplementary file 1 — Supplementary Material 1 [file 40729_2026_705_MOESM1_ESM.docx]

# SUPPLEMENTARY MATERIAL

Supplementary Table S1. Exploratory assessor-group variation and consistency based on complete item-level score sheets.

| **Endpoint** | **Assessment** | **Largest variation** | **Group comparison** | **Smallest variation / agreement** | **Group comparison** |
| --- | --- | --- | --- | --- | --- |
| Individual item score | First assessment | Soft-tissue texture; mean difference 0.338 +/- 0.081 | General practitioners vs. orthodontists | Mucosal scarring; mean difference 0.000 +/- 0.088 | Periodontists vs. general practitioners |
| Individual item score | Multidirectional assessment | Alveolar process; mean difference 0.193 +/- 0.083 | Prosthodontists vs. general practitioners | Emergence profile; mean difference 0.014 +/- 0.085 | Periodontists vs. prosthodontists |
| Total score | First assessment | Total score; mean difference 1.572 +/- 0.462 | Periodontists vs. orthodontists | Total score; mean difference 0.317 +/- 0.426 | Periodontists vs. general practitioners |
| Total score | Multidirectional assessment | Total score; mean difference 1.255 +/- 0.401 | Periodontists vs. orthodontists | Total score; mean difference 0.145 +/- 0.377 | Periodontists vs. general practitioners |

Note: Supplementary Table S1 summarizes descriptive between-group contrasts used to identify variables showing the greatest and smallest specialty-related score dispersion. These contrasts are exploratory.

.
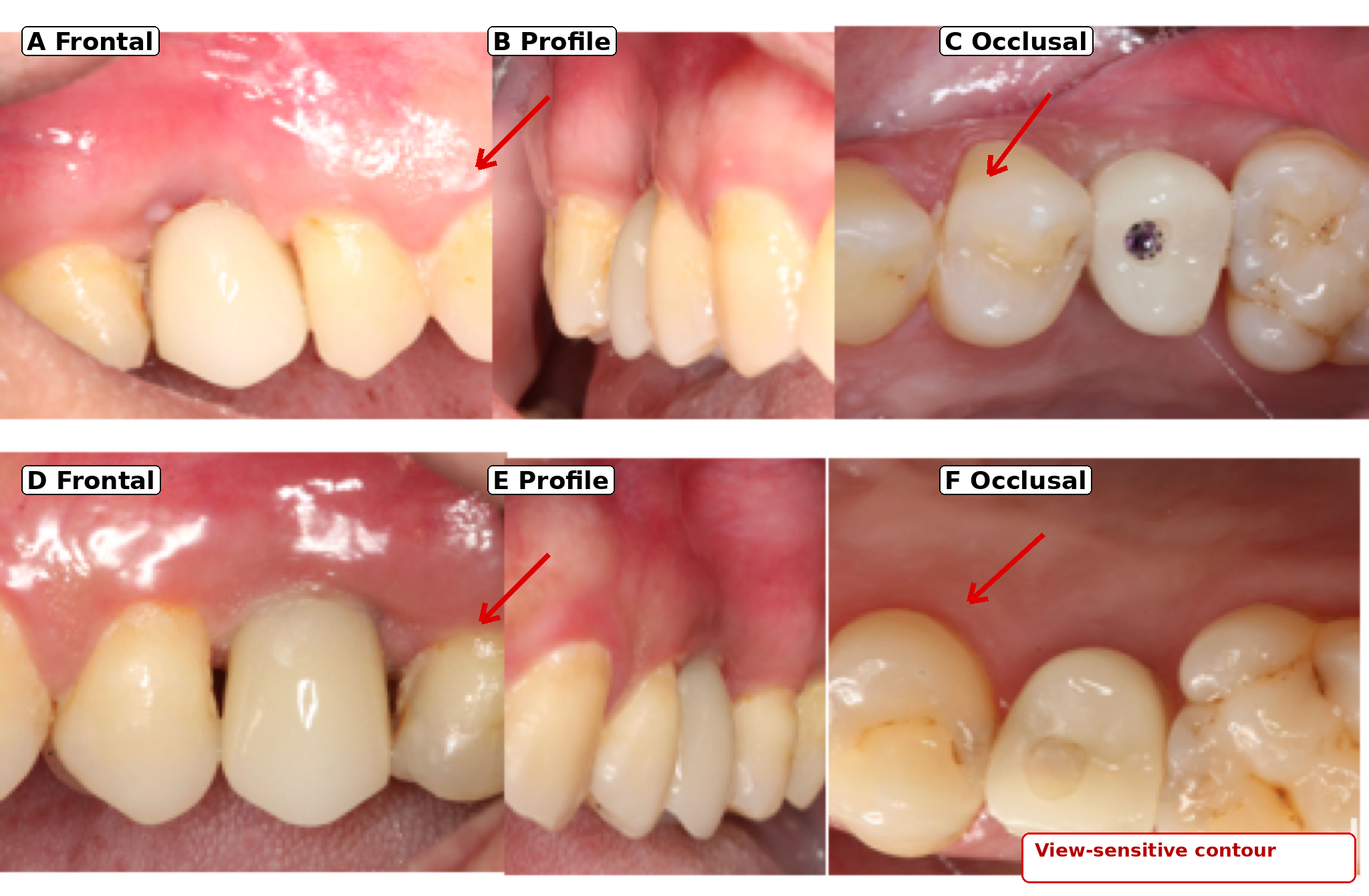


Supplementary Figure 1A. Representative clinical examples demonstrating emergence-profile and buccal-contour deficiencies that are less appreciable in frontal view but become more evident in profile and occlusal views. Panels A-C and D-F show matched frontal, profile, and occlusal views from two representative cases; arrows indicate view-sensitive contour areas.


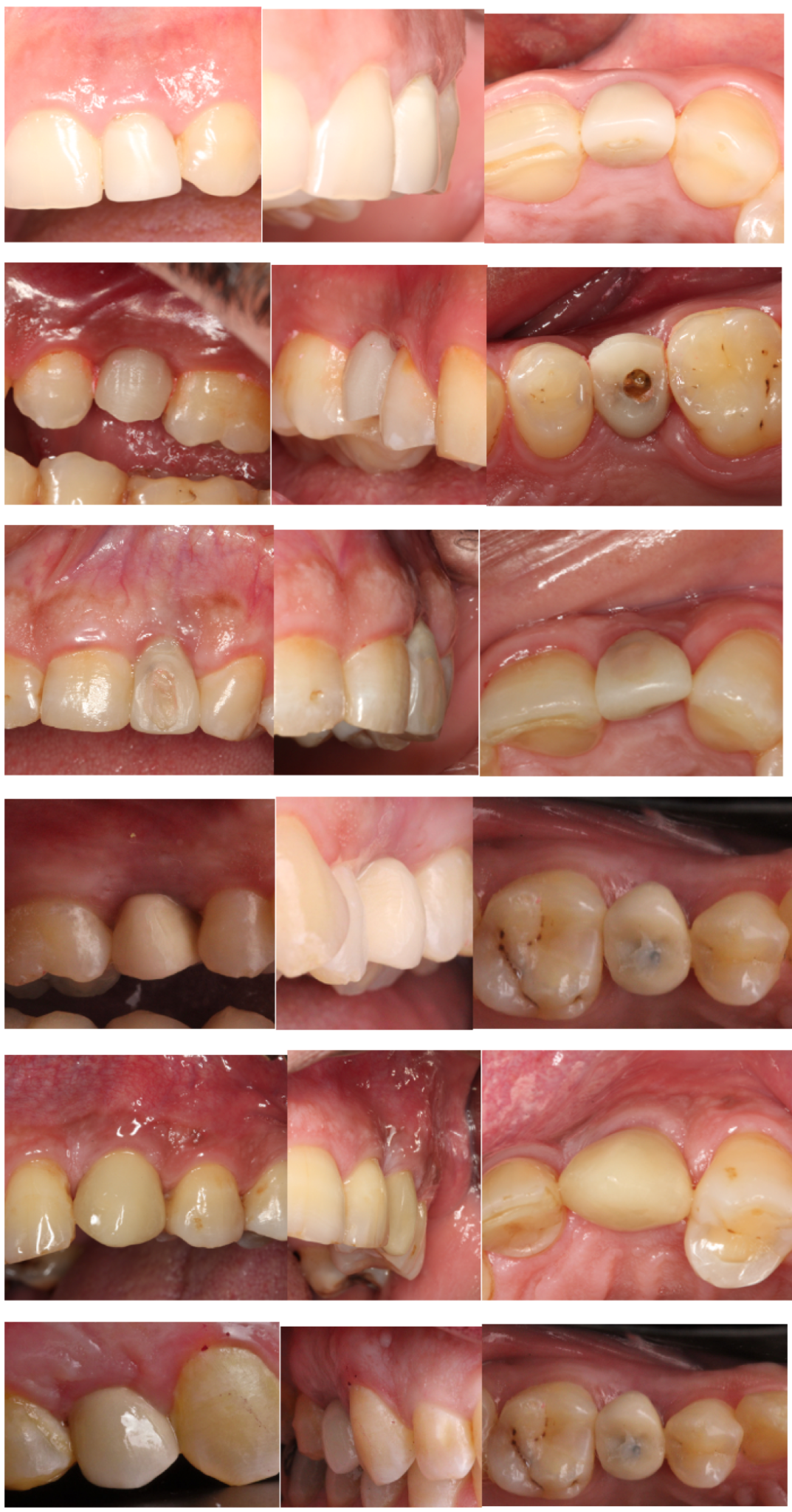


Supplementary Figure 1B. Representative clinical examples illustrating changes in surface tissue texture without corresponding mucosal scarring, supporting separation of texture and scarring as distinct E-PES variables.


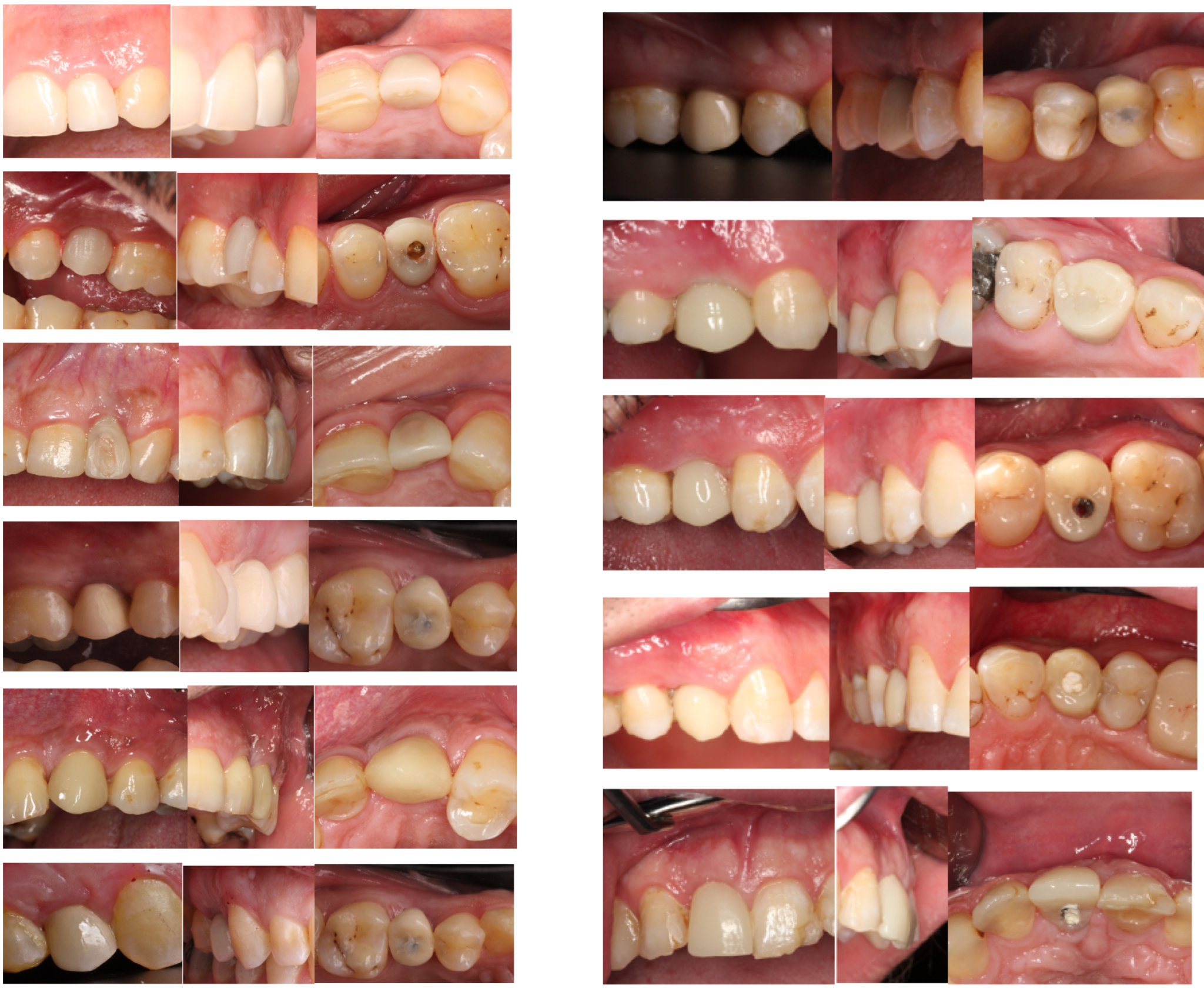


Supplementary Figure 1C. Representative clinical examples showing distinct degrees of mucosal scarring with relatively preserved surface texture features, serving as a visual scoring guide for the mucosal scarring parameter.
